# Etymologia: *Dirofilaria*

**DOI:** 10.3201/eid2002.ET2002

**Published:** 2014-02

**Keywords:** etymologia, Dirofilaria immitis, heartworm, worm, nematode

## *Dirofilaria* [diʺro-fĭ-larʹe-ə]

From the Latin *dīrus* (“fearful” or “ominous”) + *fīlum* (“thread”), *Dirofilaria* is a genus of nematodes of the superfamily Filarioidea. The first known description of *Dirofilaria* may have been by Italian nobleman Francesco Birago in 1626 in his *Treatise on Hunting*: “The dog generates two worms, which are half an arm’s length long and thicker than a finger and red like fire.” Birago erroneously identified the worms as a larval stage of another parasite, *Dioctophyme renale*. The dog heartworm was named *Filaria* by American parasitologist Joseph Leidy in 1856, and the genus was renamed *Dirofilaria* by French parasitologists Railliet and Henry in 1911.
